# Differential cytokine withdrawal-induced death sensitivity of effector T cells derived from distinct human CD8^+^ memory subsets

**DOI:** 10.1038/cddiscovery.2017.31

**Published:** 2017-05-29

**Authors:** Sasha E Larsen, Kelsey Voss, Eric D Laing, Andrew L Snow

**Affiliations:** 1 Department of Pharmacology and Molecular Therapeutics, Uniformed Services University of the Health Sciences, Bethesda, MD, USA; 2 Department of Microbiology and Immunology, Uniformed Services University of the Health Sciences, Bethesda, MD, USA

## Abstract

CD8^+^ central memory (CM) and effector memory (EM) T-cell subsets exhibit well-established differences in proliferative and protective capacity after infectious challenge. However, their relative sensitivity to apoptosis has been largely overlooked, despite the importance of programmed cell death in regulating effector T-cell homeostasis. Here we demonstrate that primary human effector T cells derived from the CD8^+^ EM subset exhibit significantly higher sensitivity to cytokine withdrawal-induced cell death (CWID), a critical intrinsic apoptosis program responsible for culling cells once an infection is cleared and interleukin-2 (IL-2) levels diminish. Interestingly, we found no differences in the expression of IL-2 or IL-2 receptor components in cells originating from either subset. Relative to CM-derived effectors, however, EM-derived T cells displayed more mitochondrial instability and greater caspase activity. Indeed, we found that heightened CWID sensitivity in EM-derived effectors coincided with higher expression of the pro-apoptotic Bcl-2 family protein BIM, both at steady state and with *de novo* induction following withdrawal of exogenous IL-2. These data point to ‘imprinted’ differences in BIM protein regulation, preserved by CD8^+^ CM and EM progeny, which govern their relative sensitivity to CWID. In addition, we detected a burst of autophagy after IL-2 withdrawal, which was better maintained in CM-derived T cells. Both subsets showed increased, equivalent CWID sensitivity upon treatment with autophagy inhibitors, suggesting sustained autophagy could preferentially protect CM-derived T cells from apoptosis. These findings offer new insight into how CM CD8^+^ T cells display superior effector cell expansion and more persistent memory responses *in vivo* relative to EM-derived T cells, based in part on decreased CWID sensitivity.

## Introduction

CD8^+^ T-cell memory constitutes an important ‘record’ of adaptive immune responses to intracellular pathogens, poised to mount more robust and efficient pathogen clearance upon re-encounter. Central memory (CM) and effector memory (EM) T-cell CD8^+^ subsets demonstrate equivalent cytotoxic activity and cytokine production upon T-cell receptor (TCR) stimulation.^1,2,3^ However, these subsets exhibit differences in longevity and protective capacity after infectious challenge.^2,3, 4,5^ CM T cells are less differentiated, exhibit self-renewal, and are longer-lived *in vivo*, contributing to enhanced engraftment after adoptive transfer compared with EM T cells.^1,2,2,4,5,6^ Previous literature suggests a greater ‘proliferative potential’ for CM T cells, giving rise to a larger effector T-cell pool, explains their superior control of viral infection. Although frequently equated with cell proliferation, total T-cell accumulation is more accurately calculated as proliferation *minus* cell death. This balance ultimately governs the magnitude and duration of an effector T-cell response. For example, secondary effectors derived from memory T cells are less sensitive to apoptosis *in vivo* after pathogen clearance than naive T-cell-derived effectors.^7^ However, despite the importance of programmed cell death in effector T-cell homeostasis, the respective apoptosis sensitivity of CM and EM T cells and their derived effectors has not been studied extensively. The continuum of T-cell memory represented by distinct subsets may also reflect a hierarchy of cell death sensitivity. Indeed, some reports have demonstrated that more terminally differentiated EM T cells possess higher caspase activity,^2^ suggesting EM T cells are ‘closer’ to a threshold for commitment to apoptosis than CM T cells.

Cytokine withdrawal-induced cell death (CWID) is the critical apoptosis program responsible for culling the majority of effector T cells, triggered by waning interleukin-2 (IL-2) levels after an infection is cleared.^8^ CWID is primarily regulated by pro- and anti-apoptotic members of the B-cell lymphoma 2 (Bcl-2) protein family.^9,10,11^ Anti-apoptotic proteins such as Bcl-2 and Bcl-xL normally help to maintain mitochondrial outer membrane integrity.^11,12^ In the absence of IL-2 receptor (IL-2R) signaling, however, pro-apoptotic BH3-only proteins such as BIM are de-repressed. Once BIM expression levels overwhelm anti-apoptotic Bcl-2 family proteins, Bax and Bak are released to form pores in the mitochondrial outer membrane, resulting in mitochondrial depolarization and caspase activation, culminating in apoptosis.^9,10,11,13^ CWID sensitivity therefore has a major role in determining which and how many T cells survive contraction and enter the memory pool, influencing secondary responses derived from distinct memory subsets.

We hypothesized that CM T cells give rise to quantitatively larger effector T-cell responses in part because of decreased apoptosis sensitivity compared with EM T cells. Here we demonstrate that primary human effector T cells derived from the CD8^+^ CM T-cell subset exhibit significantly lower sensitivity to CWID. Our data suggest that this reduced sensitivity is linked to decreased BIM induction and sustained, protective autophagy in CM-derived T cells.

## Results

**Figure 1:**
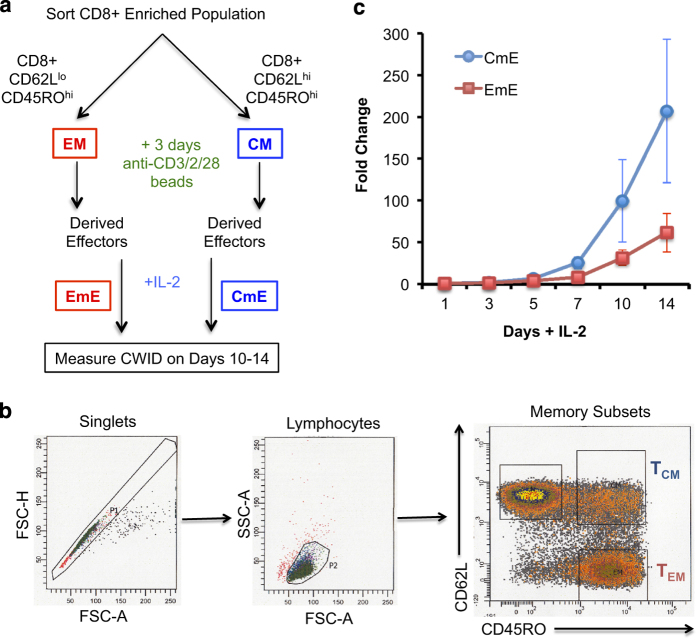
Activated human CM CD8^+^ T cells give rise to more effectors over time compared with EM T cells. (**a**) Study design: deriving effector T cells from primary human CD8^+^ CM and EM sorted subsets. (**b**) Representative example of gating/sorting strategy for CM and EM within the CD8^+^ T-cell population. (**c**) Fold change of effector T-cell expansion in IL-2 over 14 days. Data represent average expansion±S.D. of six independent donors.

**Figure 2:**
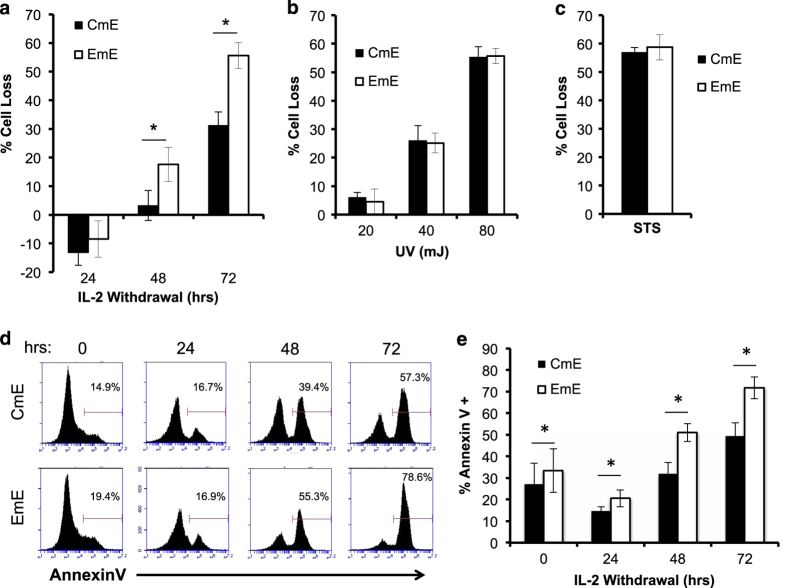
EmE are more sensitive to CWID than CmE. (**a**) Activated T cells derived from CM or EM subsets were thoroughly washed with PBS and resuspended in complete media without IL-2 as described in Materials and Methods section. On days 0–3 of IL-2 withdrawal, % cell loss was measured by PI staining and flow cytometry. Data represent % cell loss (avg±S.E.M.) for 14 individual donors. Two-way ANOVA analysis showed significant differences at 48 and 72 h; *P*<0.0001. (**b**) CmE and EmE T cells were treated with increasing doses of UV irradiation and assayed 24 h later by PI staining and flow cytometry. Data represent % cell loss (avg±S.E.M.) for three individual donors. Two-way ANOVA analysis, n.s. at all doses. (**c**) CmE and EmE T cells were treated with 2 *μ*M STS and assayed 24 h later by PI staining and flow cytometry. Data represent % cell loss (avg±S.E.M.) for three individual donors. Student’s *t-*test, n.s. (**d**–**e**) CmE and EmE T cells were stained with Annexin V on each day of IL-2 withdrawal and analyzed by flow cytometry. (**d**) Representative Annexin V staining histograms from a single donor over time. Numbers denote % Annexin V^+^ cells. (**e**) Data represent average±S.D. for four individual donors. Two-way ANOVA analysis showed CmE-EmE at 0 h *P*=0.0021, 24 h *P*=0.0033, 48 h *P*<0.0001, 72 h *P*<0.0001. Asterisks denote statistical significance.

In order to test CWID sensitivity between effector T cells derived from memory T-cell subsets, we purified CD8^+^ T cells from normal healthy human donor blood and sorted CM (CD62L^hi^ CD45RO^hi^) and EM (CD62L^lo^ CD45RO^hi^) T cells (Figures 1a and b) by FACS. Activated effector T cells were derived from each subset and cultured in media containing IL-2 for 10–14 days. As expected, donor CM T cells were consistently able to generate a larger effector population over time than EM T cells (Figure 1c). To measure CWID sensitivity of CM-derived effector T cells (CmE) *versus* EM-derived effector T cells (EmE),^14^ cells were thoroughly washed to remove all IL-2 from the cell culture medium, and cell death was monitored over 3 days of culture. EmE T cells were significantly more sensitive to CWID than CmE T cells at 48 and 72 h post IL-2 withdrawal, both by propidium iodide (PI) exclusion and Annexin V staining as indicators of late and early apoptosis commitment, respectively (Figures 2a and d–e). EmE T cells consistently demonstrated slightly higher baseline Annexin V staining for each donor tested (Figures 2d and e), suggesting they were undergoing more apoptosis at steady state and may in fact be more ‘poised to die’ after cytokine withdrawal. However, CmE and EmE demonstrated equivalent sensitivity to other intrinsic apoptotic stimuli, including UV irradiation or treatment with the pan-kinase inhibitor staurosporine (STS; Figures 2b and c). These results suggest that differential CWID sensitivity is a specific phenomenon for CmE *versus* EmE, and not indicative of a global intrinsic apoptosis defect in CmE.

**Figure 3:**
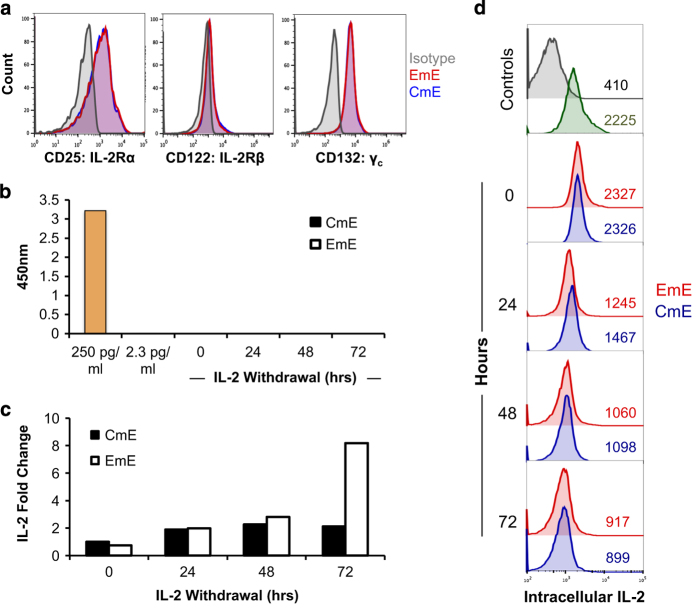
Comparable IL-2R expression and IL-2 autocrine signaling between CmE and EmE. (**a**) Representative surface staining of IL-2R chains for CmE (blue) and EmE (red) T cells *versus* isotype control (gray) by flow cytometry. (**b**) IL-2 measured in CmE and EmE T-cell supernatants from each day of IL-2 withdrawal by ELISA. Spiked IL-2 media controls (250, 2.3 pg/ml) are shown in yellow. Data represent average±S.E.M. for three individual donors. (**c**) Relative expression of IL-2 mRNA measured by qPCR during IL-2 withdrawal. IL-2 mRNA was standardized to internal control RPL13 for each sample; CmE baseline (0 h) was normalized to 1. Data are representative of four independent experiments using different donors. (**d**) Representative IL-2 intracellular flow cytometric staining for CmE (blue) *versus* EmE (red) over 0–3 days of IL-2 withdrawal, compared with isotype control (gray) and PBMC stimulated for 2 h (green). Numbers denote IL-2 MFI in each panel.

To ask whether CWID sensitivity might be tied to IL-2R expression, we compared the expression of IL-2R components and IL-2 itself between CmE and EmE. Both subsets showed equal baseline expression of all three subunits of the IL-2R (*α*, *β*, *γ*_c_) (Figure 3a), suggesting differential receptor expression does not account for differences in death sensitivity. Furthermore, IL-2 was undetectable by ELISA in cell culture supernatants on any day following withdrawal of exogenously added IL-2 (Figure 3b). Although an increase in IL-2 mRNA was detected by qPCR, we found no marked differences between CmE and EmE T cells from 0 to 48 h post exogenous IL-2 withdrawal (Figure 3c). Higher IL-2 mRNA levels were noted in EmE T cells at 72 h in some donors (Figure 3c), although any appreciable IL-2 being made at this time point was unable to rescue the majority of EmE T cells from cell death (Figure 2a). To ensure we had accounted for all IL-2 in our cultures, we also checked IL-2 expression by intracellular flow cytometry. Some retention of intracellular IL-2 was detected, which was roughly equal and progressively lower for both subsets over the course of the assay (Figure 3d). In some donors, CmE demonstrated slightly higher intracellular expression of IL-2 on day 1 of cytokine withdrawal (Figure 3d). These data suggest that although a residual, possibly recycled pool of IL-2 exists in both CmE and EmE, differential CWID sensitivity cannot be attributed to a substantial divergence in IL-2 or IL-2R expression.

**Figure 4:**
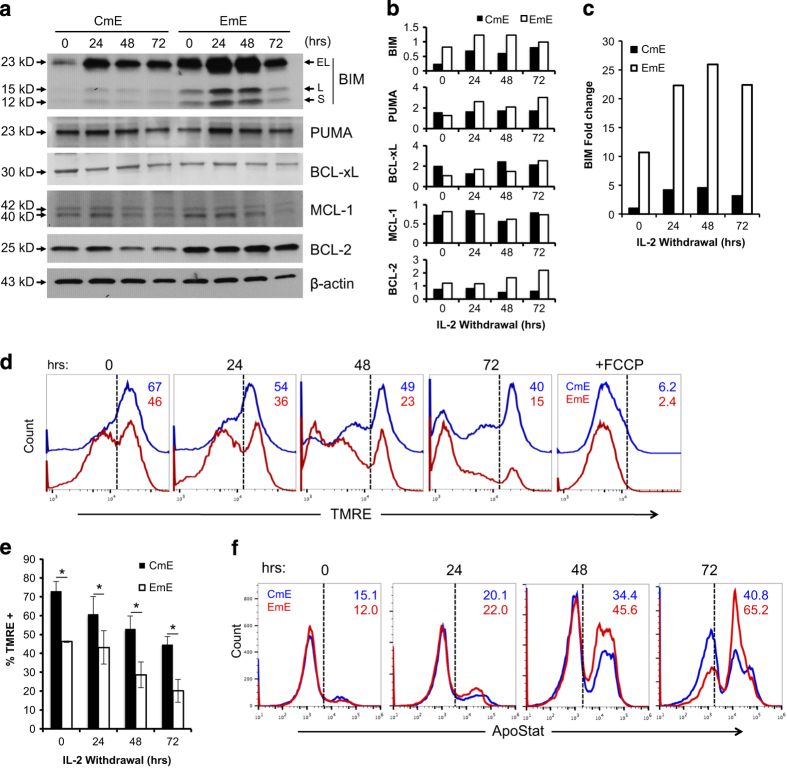
Higher BIM expression and mitochondrial instability in EmE. (**a**) Cell lysates from CmE and EmE T cells collected 0–3 days IL-2 withdrawal were immunoblotted for proteins indicated at right. Numbers at left denote specific MW for each protein; *β*-actin serves as a loading control. Data are representative of three independent experiments using different donors. (**b**) Quantification of protein expression in **a** by spot densitometry, normalized to *β*-actin. (**c**) Relative expression of BIM mRNA measured by qPCR in CmE and EmE T cells during IL-2 withdrawal. BIM mRNA was standardized to internal control RPL13 for each sample; CmE baseline was normalized to 1. Data are representative of four independent experiments using different donors. (**d **and** e**) Mitochondrial membrane potential (MOMP) was compared in CmE (blue) and EmE (red) T cells during IL-2 withdrawal using TMRE staining and flow cytometry. The uncoupling agent FCCP was used as a positive control for loss of MOMP. Numbers indicate % TMRE^hi^ cells from one representative donor. (**e**) Data represent average % TMRE positive±S.D. for three individual donors. Two-way ANOVA analysis showed CmE-EmE 0hr *P*=0.0017, 24 h *P*=0.0140, 48 h *P*=0.0027, 72 h *P*=0.0026. (**f**) Representative flow cytometric quantification of intracellular caspase activity in CmE and EmE described above (**d**) using ApoStat. Numbers denote %Apostat^+^ (active caspase) in each panel. Asterisks denote statistical significance.

We next investigated the expression of critical Bcl-2 family proteins in CD8^+^ CmE and EmE over time after IL-2 withdrawal. Strasser and colleagues previously identified BIM as the key pro-apoptotic BH3-only protein responsible for initiating CWID in activated T cells.^13^ Conversely, MCL-1 is a key anti-apoptotic Bcl-2 family protein with the highest binding affinity for BIM.^9,11,15^ Effector T cells derived from both CM and EM subsets expressed relatively similar, tapering levels of MCL-1 (appearing as a 40/42 kD doublet) during the IL-2 withdrawal time course (Figures 4a and b). In contrast, we observed substantially higher basal expression and robust induction of BIM protein and mRNA in EmE over time after IL-2 withdrawal, relative to CmE (Figures 4a and c). This marked difference in the BIM:MCL-1 ratio over time is consistent with differential CWID sensitivity observed in Figure 2a. Similarly, EmE T cells also demonstrated slightly higher expression of the pro-apoptotic protein PUMA, which also participates in CWID execution (Figures 4a and b).^16^ Although Bcl-xL expression was variable, EmE T cells did consistently demonstrate slightly higher expression of BCL-2 (Figures 4a and b), which may help this subset manage the considerably higher basal expression of BIM.

An increase in pro *versus* anti-apoptotic Bcl-2 family proteins ultimately governs the disruption of mitochondrial membrane integrity required for intrinsic apoptosis. We therefore asked whether higher basal expression of BIM in EmE corresponded with a reduction in mitochondrial membrane potential *versus* CmE. Indeed, at baseline and during IL-2 withdrawal, EmE T cells demonstrated significantly lower retention of the mitochondria-specific dye tetramethylrhodamine, ethyl ester (TMRE) compared with CmE T cells (Figures 4d and e). In addition, EmE T cells displayed higher pan-caspase activity during days 2–3 of cytokine withdrawal (Figure 4f). Again, these data suggest that EmE T cells may be ‘poised’ for more rapid CWID than CmE, via enhanced BIM expression and mitochondrial instability.

**Figure 5:**
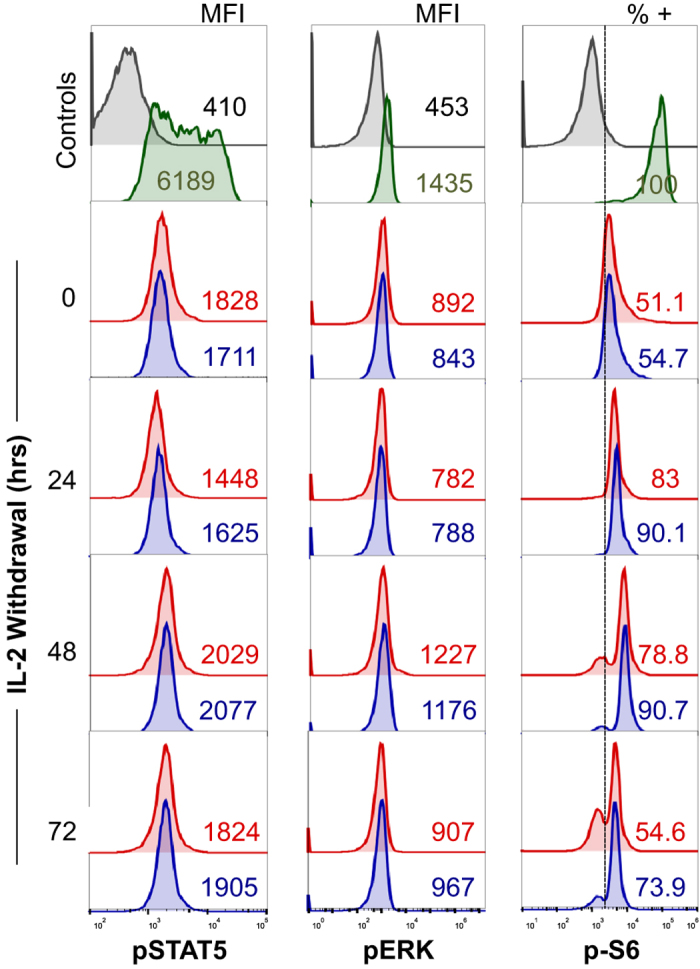
Differential phosphorylation of S6 between CmE and EmE T cells during IL-2 withdrawal. Representative intracellular flow cytometric staining for phospho-STAT5 (left), phospho-ERK^39^ and phospho-S6^40^ in CmE (blue) and EmE (red) T cells during IL-2 withdrawal; isotype control (gray) and PBMC stimulated for 2 h with IL-2 and PMA (green) serve as controls. Numbers in each panel denote MFI for phospho-STAT5 and phospho-ERK signal, or % p-S6^+^ cells in CmE and EmE.

As IL-2R and IL-2 expression was comparable in both subsets, we next utilized intracellular FACS staining to probe key IL-2R signaling pathways for discrepancies between CmE and EmE that could help to explain the significant difference in BIM expression and CWID (Figure 2a). CmE and EmE showed low, roughly equal levels of phospho-STAT5, a critical proximal indicator of IL-2 signaling (Figure 5). We also noted equivalent phosphorylation of ERK, a representative distal kinase, which fluctuated slightly over the course of IL-2 withdrawal in both subsets (Figure 5). Diminished levels of both phospho-STAT5 and phospho-ERK mirrored decreasing levels of intracellular IL-2 (Figure 3d) noted as CWID progressed.

We also interrogated the activity of the mechanistic target of rapamycin (mTOR) via phosphorylation of one of its primary targets, ribosomal protein S6. mTOR regulates cell growth and metabolism by integrating multiple growth factor and nutrient signals from within and around the cell, including IL-2.^17,18, 19,20^ In our CWID assay, we hypothesized that as nutrients are ostensibly depleted from the culture media in the absence of IL-2, mTOR activity would decrease over time.^21^ However, effectors derived from both memory subsets displayed a burst of S6 phosphorylation 24 h after cytokine withdrawal (Figure 5). Importantly, a greater proportion of CmE demonstrated a sustained phospho-S6 signal over time compared with EmE (Figure 5). These results reveal a difference in sustained mTOR signaling induced after 24 h of IL-2 withdrawal, congruent with the time point at which CWID sensitivity diverges between CmE and EmE.

The abrupt increase in S6 phosphorylation noted in effector T cells under conditions of growth factor withdrawal seemed counterintuitive. However, mTOR is also responsive to increasing levels of free cellular amino acids, a major product of autophagy.^22,23,24^ Autophagy is a catabolic program induced to adapt to starvation conditions and maintain cellular homeostasis. Autophagy has long held a seemingly conflicting influence on both cell survival and programmed cell death.^25^ The contribution of autophagy in promoting or delaying programmed cell death during cytokine withdrawal in CD8^+^ T cells is unknown. We hypothesized that the observed burst of S6 phosphorylation beginning on day 1 of CWID reflected feedback mTOR activation via autophagy.

**Figure 6:**
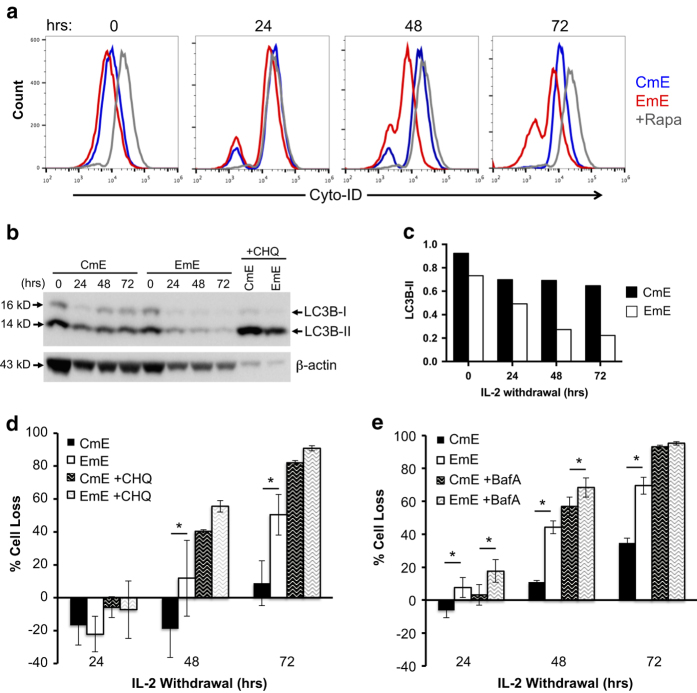
CmE exhibit greater protective autophagy in response to IL-2 withdrawal. (**a**) Representative intracellular flow cytometric Cyto-ID staining of autophagic vesicles in CmE (blue) and EmE (red) T cells during IL-2 withdrawal. The mTOR inhibitor rapamycin (gray) serves as a positive control. (**b**) Cell lysates from CmE and EmE T cells collected 0–3 days IL-2 withdrawal were immunoblotted for LC3B and *β*-actin. LC3B-I and LC3B-II (lipidated form) bands are indicated at right. Data are representative of three independent experiments. (**c**) Quantification of LC3B-II expression in (**a**) by spot densitometry, normalized to *β*-actin. (**d **and **e**) CmE and EmE T cells washed and resuspended in complete media without IL-2 as previously described were treated with 10 *μ*M CHQ or ddH_2_0 (**b**), or 10 *μ*M BafA or DMSO (**c**). On days 0–3 of IL-2 withdrawal, % cell loss was measured by PI staining and flow cytometry. Data represent average % cell loss±S.E.M. of two (**b**) or three (**c**) independent donors. Two-way ANOVA analysis for (**b**) showed CmE-EmE 24 h n.s., CmE+CHQ-EmE+CHQ 24 h n.s., CmE-EmE 48 h *P*=0.0018, CmE+CHQ-EmE+CHQ 48 h n.s., CmE-EmE 72 h *P*=0.0003, CmE+CHQ-EmE+CHQ 72 h n.s. Two-way ANOVA analysis for **c** showed CmE-EmE 24 hr *P*=0.0031, CmE+BafA-EmE+BafA 24 h *P*=0.0021, CmE-EmE 48 h *P*<0.0001, CmE+BafA-EmE+BafA 48 h *P*=0.0122, CmE-EmE 72 h *P*<0.0001, CmE+BafA-EmE+BafA 72 h n.s. Asterisks denote statistical significance.

To test this hypothesis, we next investigated whether CmE and EmE exhibited differential autophagic activity following IL-2 withdrawal by staining cells with Cyto-ID, a dye that labels autophagic vacuoles.^26^ At baseline, CmE T cells exhibited slightly higher levels of autophagy compared with EmE T cells (Figure 6a). Both CmE and EmE exhibited a burst of autophagic activity 24 h after IL-2 withdrawal, comparable to levels noted with the mTOR inhibitor rapamycin, which served as a positive control (Figure 6a). However, only CmE retained this high level of autophagy on days 2 and 3 of IL-2 withdrawal, whereas Cyto-ID staining waned in EmE T cells (Figure 6a). Accumulation of the autophagosome protein marker LC3B-II followed a similar pattern, with sustained LC3B-II expression (relative to *β*-actin expression) specifically noted in CmE during IL-2 withdrawal (Figures 6b and c). This temporal discrepancy in sustained autophagy (and S6 phosphorylation) closely correlated with the difference in CWID in CmE *versus* EmE, which began to manifest on day 2 of IL-2 withdrawal (Figure 2a). In order to determine if higher autophagic flux was in fact protecting CmE T cells from CWID, we pre-treated both subsets with the autophagy inhibitors chloroquine (CHQ) or bafilomycin A (BafA) and analyzed changes in apoptosis sensitivity over time. In the presence of IL-2, neither drug induced significant toxicity on its own (data not shown). However, both CHQ and BafA treatment increased CWID sensitivity of both effector subsets (Figure 6d). As expected, blockade of autophagic flux with CHQ resulted in increased LC3B-II accumulation that was more pronounced in CmE (Figure 6b). More importantly, autophagy inhibition eliminated the difference in CWID sensitivity between CmE and EmE (Figure 6e). These data illuminate a protective role for autophagy that delays apoptosis in effector CD8^+^ T cells following cytokine withdrawal. Furthermore, our data suggest that sustained autophagy helps protect more CM-derived effectors from CWID.

## Discussion

Adoptive transfer studies in mice have assessed the protective capacity of CD8^+^ memory T-cell subsets in response to pathogen rechallenge.^1,2,3,4,6,27,28,29,30^ Less differentiated memory subsets (for example, CM) consistently clear pathogen more quickly, presumably via generation of a larger effector pool.^1,2,3,4, 5^ Effector T-cell accumulation, however, is ultimately determined by a balance of both proliferation and cell death. CWID is the primary cell death program that culls the majority of activated effector T cells after clearance of pathogen. However, the relative sensitivity of memory-derived effector T cells to CWID has not been established, despite its major influence on T-cell accumulation and homeostasis. We hypothesized that greater effector T-cell accumulation derived from CM *versus* EM T cells was indicative, in part, of differential CWID sensitivity.

Indeed, our findings demonstrate that effectors derived from CM T cells are significantly and specifically less sensitive to CWID than EM-derived effectors. This disparity in CWID sensitivity was characterized by quantitative differences in multiple apoptosis markers, including Annexin V binding, mitochondrial membrane potential, caspase activation and PI incorporation. Both subsets demonstrated equal expression of IL-2R signaling components and no measurable, endogenous IL-2 secretion. In line with recent studies,^31^ we did detect some intracellular IL-2 retained in T cells after IL-2 withdrawal, which waned in both subsets over time. However, we suspect signaling from recycled IL-2/IL-2R complexes in our system was minimal, considering downstream readouts of IL-2R signaling (phospho-STAT5, phospho-ERK) remained relatively low after IL-2 withdrawal, and were comparable between CmE and EmE (Figure 5).

Despite no appreciable differences in the IL-2 signaling cascade, we did uncover higher basal expression and induction of the pro-apoptotic protein BIM after IL-2 withdrawal in EmE compared with CmE. These data suggest ‘imprinted’ differences in BIM protein regulation, preserved between CD8^+^ CM and EM progeny, that predispose EmE to increased cell death sensitivity through cytokine withdrawal. Indeed, EmE also displayed decreased mitochondrial membrane integrity, and increased caspase activity induced by IL-2 withdrawal, suggesting EmE are more predisposed for CWID. These findings are in line with previous reports noting that EmE are more terminally differentiated and prone to apoptosis in mice.^2^

In contrast to STAT5 and ERK signals, we noted a burst of S6 phosphorylation 24 h after IL-2 withdrawal in both effector subsets, which was better sustained over time in CmE *versus* EmE. These data suggested a restoration of mTOR signaling in the first 24 h of our time course, despite the absence of IL-2 and fresh media. We hypothesized this signal could be derived from induction of autophagy, an important catabolic program that consumes old/damaged cellular components and organelles to recover and replenish amino acid reserves, particularly in response to starvation or growth factor withdrawal.^22,23,24^ Indeed, autophagy directly triggers mTOR reactivation under prolonged starvation conditions.^24^ Autophagy is a complex process that can promote survival and differentiation in certain cell contexts, or contribute directly to programmed cell death in others.^25,32,33,34,35^ Our data specifically highlight a pro-survival function for autophagy in CD8^+^ memory-derived effector T cells during CWID, consistent with many reports confirming a cytoprotective role for controlled autophagy in lymphocytes.^36^ CmE demonstrated higher, sustained accumulation of LC3B-II and autophagic vacuoles after IL-2 withdrawal, correlating with lower CWID sensitivity. When autophagic flux was blocked using CHQ or BafA, both CmE and EmE displayed increased sensitivity to CWID, suggesting some amount of autophagy protects either subset from rapid apoptosis. More importantly, autophagy blockade equalized CWID sensitivity between subsets, suggesting CmE T cells can sustain more protective autophagy than EmE and delay death after cytokine withdrawal. Our findings offer new insight into why CM-derived T cells display superior effector cell expansion and protective responses *in vivo* relative to EM-derived T cells, beyond simple proliferation capacity.

The relationship between autophagy and apoptosis is highly complex, and more work is required to understand how autophagy governs the accumulation of pro-apoptotic Bcl-2 family proteins that trigger CWID in effector T cells. For example, CmE may preferentially be able to degrade death-inducing levels of BIM via autophagy, as some recent studies have demonstrated that autophagy can degrade cell death-associated proteins in order to promote survival.^37^ It will be important to test if autophagy inhibition boosts the expression of BIM and other pro-apoptotic proteins (for example, PUMA) in effector T cells during IL-2 withdrawal, particularly in CmE. Alternatively, sustained autophagy in CmE may help to clear damaged mitochondria that initiate intrinsic apoptosis. Additional comparisons of T-cell subsets may help further unravel the intricacies of autophagy and its relationship to Bcl-2 family protein expression, apoptosis sensitivity and memory T-cell functions.

Although autophagy is induced by TCR stimulation, activated effector T cells *in vivo* undergo minimal amounts of autophagy during the expansion phase. However, rapid upregulation of autophagy is observed in effector T cells just after pathogen is cleared and before the contraction phase begins.^34^ In fact, this rapid switch to autophagy is critical for the differentiation and survival of memory T cells *in vivo*; mice lacking Atg5 or Atg7 mount a robust effector CD8^+^ T-cell response to viral infection, but are compromised in their ability to generate memory T cells.^34^ Our study complements and extends this paradigm by demonstrating a novel setpoint for autophagy induction that distinguishes human CM- and EM-derived effectors and their relative susceptibility to CWID. This imprinted, differential balance between protective autophagy and CWID, preserved in memory-derived effectors, likely influences both the size and duration of the recall response from distinct memory T-cell subsets.

## Materials and methods

### Isolation, activation and culture of primary human CD8^+^ T cells

Blood from anonymous healthy donors (buffy coats) was generously provided by Dr. Michael Lenardo and the National Institutes of Health Blood Bank. PBMC were isolated using Ficoll density gradient centrifugation, and CD8^*+*^ T cells were purified from PBMC using the EasySep Human CD8^*+*^ T-cell enrichment kit (Stem Cell Technologies, Vancouver, BC, Canada). CD8^+^ T cells (>95% purity) were stained for 30 min on ice with anti-CD45RO-APC and anti-CD62L-FITC antibodies (BioLegend, San Diego, CA, USA). Memory subsets were sorted on a BD FACSAria cell sorter (BD Biosciences, San Jose, CA, USA); CM T cells were gated as CD45RO^hi^ and CD62L^hi^, EM T cells were gated as CD45RO^hi^ and CD62L^lo^. Sorted subsets were activated 1:1 with beads coated with anti-CD3/CD2/CD28 antibodies (Human T-cell Activation/Expansion Kit, Miltenyi Biotec Inc, San Diego, CA, USA) in RPMI 1640 (Thermo Fisher Scientific, Waltham, MA, USA)+10% fetal calf serum (FCS) (Sigma-Aldrich, St Louis, MO, USA) and 1% penicillin/streptomycin (Lonza, Basel, Switzerland) for 3 days. Activated T cells were washed in PBS and subsequently cultured in media as described above with 100 U/ml rIL-2 (PeproTech, Rocky Hill, NJ, USA) at 1×10^6^ cells/ml for ≥10 days, changing media every 3 days.

### Apoptosis assays and flow cytometry

CWID assays were performed as previously described.^38^ Briefly, expanded activated effector T cells (days 10–12) were washed 3X with PBS and resuspended in fresh complete media without rIL-2. These cells were then plated in triplicate wells at 7.5×10^5^ cells/ml in 96-well plates. To investigate autophagy, some cells were treated with 10 *μ*M CHQ, 50 nM BafA (Sigma-Aldrich) or DMSO solvent control on day 0. To test sensitivity to other intrinsic death stimuli, some cells were treated with 2 *μ*M STS (Sigma-Aldrich) or subjected to 20 000–80 000 mJ/cm^2^ UV irradiation using a Stratalinker UV crosslinker (Stratagene, Agilent Technologies, Santa Clara, CA, USA). On days 0–3 of IL-2 withdrawal±inhibitor treatment, or 24 h post STS or UV irradiation, cells were stained with 5 *μ*g/ml propidium iodide (Sigma-Aldrich) and collected for constant time on an Accuri C6 flow cytometer (BD Biosciences). Cell death was quantified as percentage cell loss=(1–(number of viable cells (treated)/number of viable cells (untreated)))×100. For some assays, T cells were stained with Annexin V-FITC (BioLegend) on each day of withdrawal. Surface expression of IL-2 receptor components was assessed using anti-CD25-APC (IL-2R*α*), anti-CD122-PE (IL-2R*β*) and anti-CD132-FITC (common-*γ* chain receptor) antibodies (BioLegend). Analysis of caspase activity between subsets was performed using FITC-conjugated pan-caspase inhibitor ApoStat (R&D Systems, Minneapolis, MN, USA) according to the product protocol. Mitochondrial membrane potential was assessed by staining 5×10^6^ cells with a final concentration of 100 nM TMRE (Sigma-Aldrich) in PBS for 25 min at 37 °C, followed by flow cytometry analysis. Autophagy activity was monitored on each day of IL-2 withdrawal using the Cyto-ID kit (Enzo Life Sciences Inc, Farmingdale, NY, USA) according to the manufacturer’s protocol, followed by flow cytometry analysis. Intracellular staining was performed using the FOXP3 intracellular staining kit (Thermo Fisher Scientific) per protocol using anti-phospho(235/236)-S6-AlexaFluor 488 (Cell Signaling Technology, Danvers, MA, USA); anti-IL-2-APC; anti-phospho(T202/Y204)-ERK-PercP-Cy5.5; and anti-phospho(Y694)-STAT5-AlexaFluor 647 (BD Biosciences). All non-sorting flow cytometric assays were performed on an Accuri C6 flow cytometer (BD Biosciences).

### Western blotting

Expanded effector T cells (1×10^6^ per time point) deprived of IL-2 for 0–3 days were washed in cold PBS, and lysed in 1% Nonidet P-40 (NP-40) lysis buffer (50 mM Tris (pH 7.4), 150 mM NaCl, 0.5 mM EDTA, 1% NP-40, 0.5% sodium deoxycholate, 1 mM Na_3_VO_4_, 1 mM NaF) containing complete protease inhibitors (Roche Diagnostics Corp., Indianapolis, IN, USA) for 30 min on ice. For LC3 blotting, cells were lysed in 10 mM Tris-Cl, pH 6.8, 69 mM NaCl, 0.5 mM EGTA, 0.5% Triton X-100, 5% glycerol containing complete protease inhibitors (Roche Diagnostics Corp.) for 30 min on ice. Cleared lysates were boiled in 2x reducing sample buffer and resolved on Any kD SDS-PAGE gels (Bio-Rad Laboratories, Hercules, CA, USA). Proteins were transferred to nitrocellulose or PVDF (for LC3) on a Trans-Blot Turbo system (Bio-Rad), blocked with 2% Tropix I-Block (Thermo Fisher Scientific) or 6% milk (Bio-Rad) in TBS/0.1% Tween, and probed with the following Abs: anti-BIM (Enzo Life Sciences Inc); anti-PUMA (Cell Signaling Technology); anti-Bcl-xL, anti-Mcl-1, anti-Bcl-2 (BD Biosciences); anti-LC3B and anti-*β*-actin (Sigma-Aldrich). Bound Abs were detected using HRP-conjugated secondary Abs (Southern Biotech, Birmingham, AL USA; Agilent Technologies) and ECL (Thermo Fisher Scientific). Densitometry analysis was performed using ImageJ software (www.imagej.net). Full blots are included as Supplementary Information.

### ELISA

Secreted IL-2 was measured in the cell culture supernatant on days 0–3 of CWID using the human Ready-Set-Go IL-2 ELISA kit (Agilent Technologies). ELISA plates were read using a Synergy H1 Hybrid Reader (BioTek, Winooski, VT, USA); concentrations of IL-2 (pg/ml) were calculated using Gen5 data analysis software (BioTek).

### Quantitative PCR

RNA was isolated from CM- or EM-derived effector T cells from 4 donors for each day of IL-2 withdrawal using QIAshredder and RNEasy mini plus columns (Qiagen, Hilden, Germany). cDNA was prepared from 300 ng RNA using the High Capacity cDNA Reverse Transcription Kit (Applied Biosystems, Thermo Fisher Scientific). Maxima SYBR Green/ROX qPCR Master Mix (Thermo Fisher Scientific) was used for subsequent PCR with specific primers against BIM (for: 5′-CTGAGTGTGACCGAGAAGGT-3′, rev: 5′-TGTGGCTCTGTCTGTAGGGA-3′), IL-2 (Bio-Rad PrimePCR SYBR Green Assay, Bio-Rad Laboratories Inc., Hercules, CA, USA: IL-2, Human) and RPL13 (for: 5′-GAATGGCATGGTCTTGAAGCC-3′, rev: 5′-GGGAATGTGCTGTTTCCATGG-3′) as a reference control. Analysis was conducted on a StepOnePlus Real-Time PCR System (Applied Biosystems, Thermo Fisher Scientific).

### Statistics

*In vitro* cell death assays were evaluated using two-way ANOVA (*α*=0.05) with Sidak correction for multiple comparisons, or Student’s* t-*test where appropriate. All statistical analyses were performed using GraphPad PRISM software (GraphPad Inc, La Jolla, CA, USA). Error bars are defined in the figure legends as ±S.E.M. or ±S.D. Asterisks denote statistical significance; *P*-values are reported in figure legends.
