# Supplementary figures and images for: Differential cytokine withdrawal-induced death sensitivity of effector T cells derived from distinct human CD8+ memory subsets
Source: Cell Death Discov. 2017 May 29;3:17031–. doi: 10.1038/cddiscovery.2017.31 (PMC5447130; doi:10.1038/cddiscovery.2017.31)

# CDDISCOVERY.2017.31 Supplementary Information: Full blots

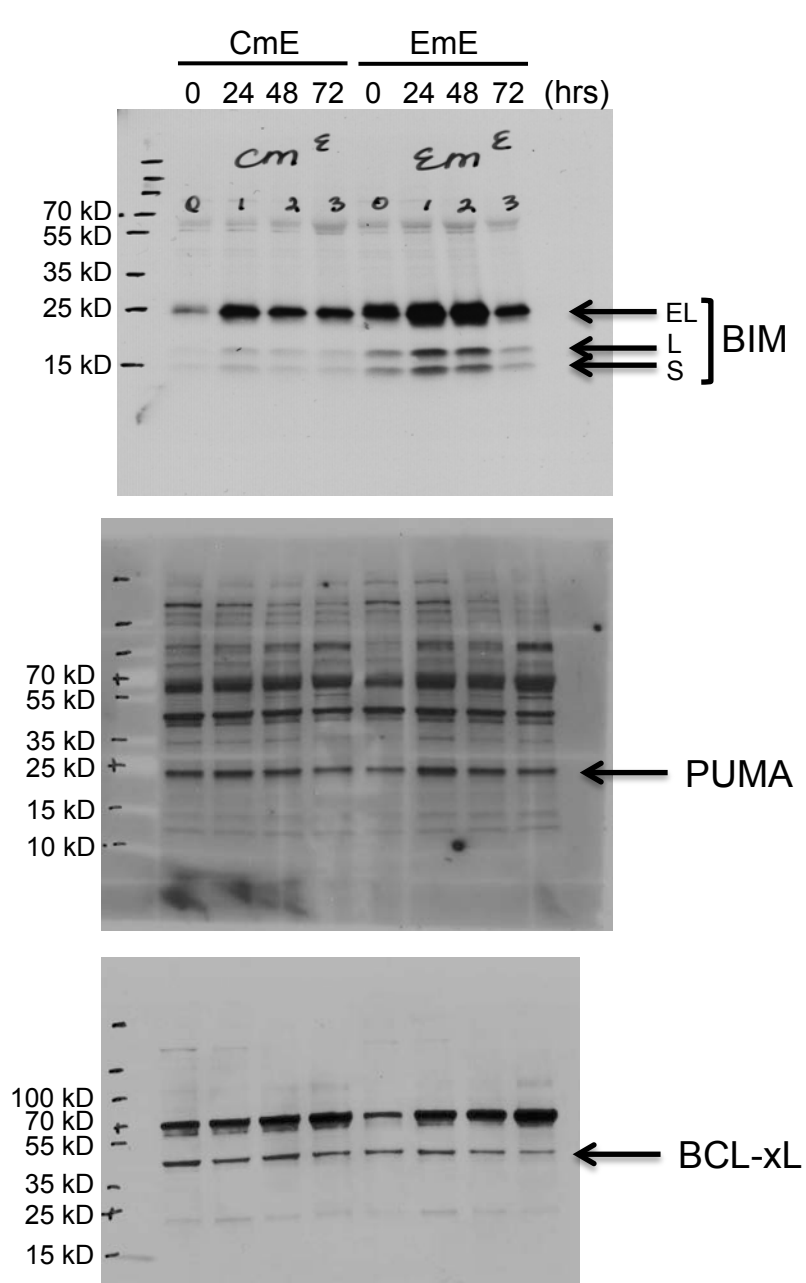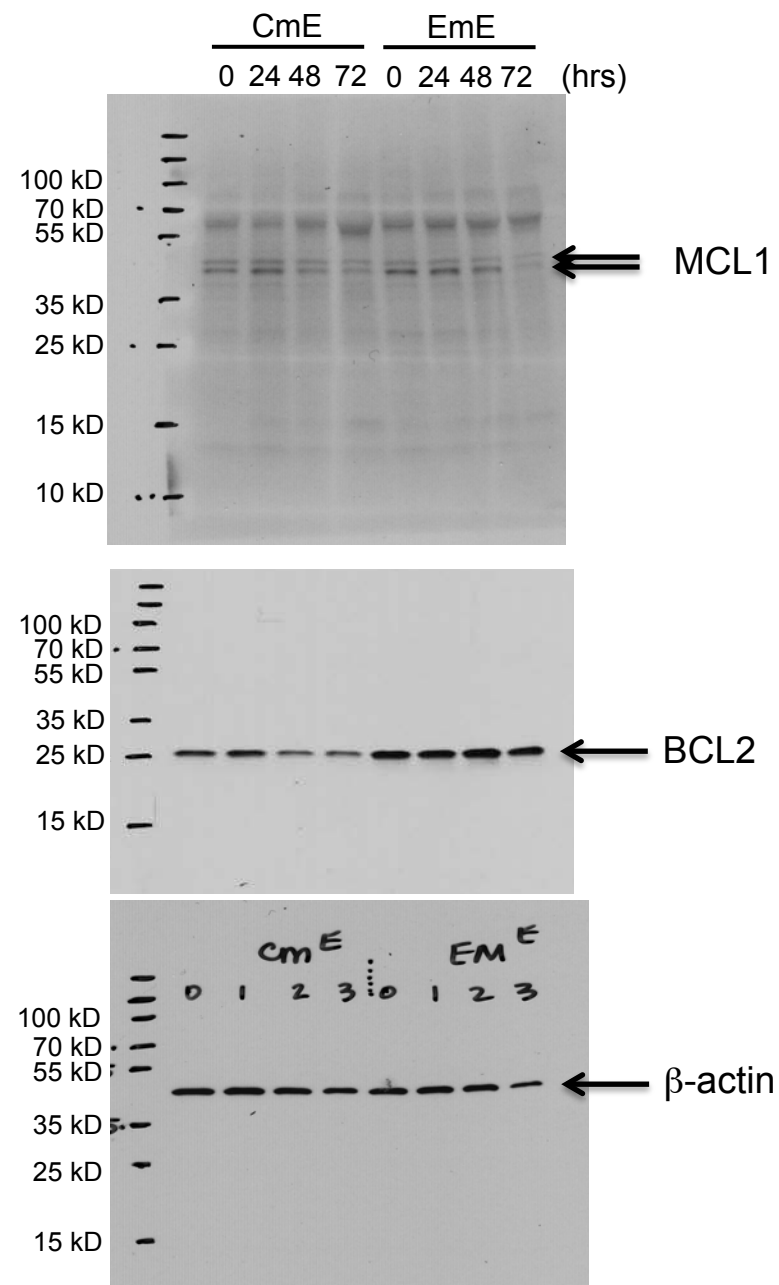

Supplement: Supplementary Information [file cddiscovery201731-s1.pdf]
